# Influence of Light Intensity and Spectrum on Duckweed Growth and Proteins in a Small-Scale, Re-Circulating Indoor Vertical Farm

**DOI:** 10.3390/plants11081010

**Published:** 2022-04-07

**Authors:** Finn Petersen, Johannes Demann, Dina Restemeyer, Hans-Werner Olfs, Heiner Westendarp, Klaus-Juergen Appenroth, Andreas Ulbrich

**Affiliations:** 1 Faculty of Agricultural Sciences and Landscape Architecture, University of Applied Sciences Osnabrück, Am Krümpel 31, 49090 Osnabrück, Germany; johannes.demann@hs-osnabrueck.de (J.D.); dina.restemeyer@hs-osnabrueck.de (D.R.); h-w.olfs@hs-osnabrueck.de (H.-W.O.); h.westendarp@hs-osnabrueck.de (H.W.); a.ulbrich@hs-osnabrueck.de (A.U.); 2 Matthias-Schleiden-Institute–Plant Physiology, University of Jena, Dornburger Str. 159, 07743 Jena, Germany; klaus.appenroth@uni-jena.de

**Keywords:** Lemnaceae, *Lemna minor*, *Wolffiella hyalina*, red/blue ratio, standardized production, yield, light quality, light quantity, controlled environment

## Abstract

Duckweeds can be potentially used in human and animal nutrition, biotechnology or wastewater treatment. To cultivate large quantities of a defined product quality, a standardized production process is needed. A small-scale, re-circulating indoor vertical farm (IVF) with artificial lighting and a nutrient control and dosing system was used for this purpose. The influence of different light intensities (50, 100 and 150 µmol m^−2^ s^−1^) and spectral distributions (red/blue ratios: 70/30, 50/50 and 30/70%) on relative growth rate (RGR), crude protein content (CPC), relative protein yield (RPY) and chlorophyll a of the duckweed species *Lemna minor* and *Wolffiella hyalina* were investigated. Increasing light intensity increased RGR (by 67% and 76%) and RPY (by 50% and 89%) and decreased chlorophyll a (by 27% and 32%) for *L. minor* and *W. hyalina*, respectively. The spectral distributions had no significant impact on any investigated parameter. *Wolffiella hyalina* achieved higher values in all investigated parameters compared to *L. minor*. This investigation proved the successful cultivation of duckweed in a small-scale, re-circulating IVF with artificial lighting.

## 1. Introduction

The term duckweed comprises 36 species [1,2] of 5 genera, belonging to the family of Lemnaceae Martinov [3,4]. They are characterized, amongst other aspects, by their fast growth rate [5,6], high nutrient uptake capacity [7,8] as well as by their edibility [9,10] and variability of nutritional values influenced by cultivation conditions [11,12]. Those are key aspects for further use in human and animal nutrition, biotechnology or wastewater treatment.

In order to continuously produce large quantities of biomass with a defined quality (e.g., for human nutrition), a standardized cultivation process is necessary. One possible solution in the future might be the cultivation of duckweed in re-circulating (also described as closed) indoor vertical farms (IVF) with artificial lighting. By stacking several layers of cultivation areas above each other, the land utilization efficiency is increased [13,14]. When operating an IVF in a controlled environment, it is possible to regulate plant-relevant abiotic factors, e.g., nutrient composition and concentration, light intensity and spectrum, photoperiod, the temperature of water and air, water flow rate or humidity according to the grower’s demand. Resources, such as nutrients, water and pesticides, can be used efficiently. This can positively affect the quantity and quality of the crops. Additionally, the use of IVFs will allow year-round crop production, even in areas with short growing seasons or unfavorable climatic conditions [13,14,15,16]. One shortcoming of this cultivation technology is the relatively high energy input, e.g., the production of one kg of curled lettuce required 7–9 kWh of electric energy [14].

However, closed hydroponic systems are already successfully used to cultivate different crops in large quantities. This includes tomatoes, cucumbers, peppers, different leafy greens, strawberries and even rice or maize [17]. The advantages of closed hydroponic systems compared to conventional farming are enormous, as up to 85% of fertilizers and 90% of water can be saved, while a productivity increase of up to 250% is possible [18]. The water and nutrient use efficiency of tomatoes cultivated in a closed hydroponic system was 23% higher compared to an open system in both cases [19]. The water use efficiency for tomatoes cultivated in a closed system in The Netherlands was 66 kg of yield per cubic meter of water applied [18]. Another study described zero discharge of nutrients and pesticides to the environment in the production of sweet peppers and autumn cucumber in a closed hydroponic system [20].

In order to also achieve an efficient system for duckweed cultivation, all necessary abiotic factors must be evaluated. Two of these abiotic factors are light intensity and the spectral light distribution. In nature, *Lemnaceae* grow in sunny as well as in shaded habitats, but the latter habitats are favorable due to lower light intensities and less extreme temperatures [21]. The plant’s reaction to different light intensities is dependent on the species and abiotic factors, such as nutrients or temperature, while the light spectrum is another important parameter [22]. *Wolffia arrhiza* cultivated in steady-state conditions with blue light showed higher protein and chlorophyll contents compared to red light [22]. Increasing light intensities slightly increased the relative growth rates (RGRs) of *Lemna gibba* [23] and *Lemna aequinoctialis* [24]. Very high intensities, however, lead to light saturation. Light intensities above this point will not increase the photosynthetic activity of the plant and could lead to damages due to oxygen stress (photoinhibition). The light saturation point depends on factors such as temperature and varies for different duckweed species. A light saturation of 342 µmol m^−2^ s^−1^ for *L. minor* [25] and of 400 µmol m^−2^ s^−1^ for *L. minor* and *Lemna minuta* were observed [26], while *Landoltia punctata* (formerly *Spirodela punctata*) reached light saturation between 600 and 1200 µmol m^−2^ s^−1^ at 30 °C [27]. Considering the cost of artificial lighting, an optimum of 110 µmol m^−2^ s^−1^ was obtained for *L. aequinoctialis* [24].

The aim of our research was to evaluate the influence of different light intensities and spectral distributions on the RGR, crude protein content (CPC) and relative protein yield (RPY) in the duckweeds *L. minor* and *W. hyalina* when cultivated in a small-scale, re-circulating, aquatic IVF. Additionally, for both species, the chlorophyll a content was determined as a plant cultivation indicator. We selected clones of these two species because they showed good performance in earlier experiments concerning growth rates and protein contents [9].

## 2. Materials and Methods

### 2.1. Indoor Vertical Farm

Two duckweed species, *Lemna minor* L. (clone 9441; Germany) and *Wolffiella hyalina* Delile Monod (clone 9525; India), were chosen for the experiments due to their fast growth rates and high protein contents [28]. The plant material was obtained from the Duckweed Stock Collection of the Department of Plant Physiology, University of Jena, Germany.

**Figure 1:**
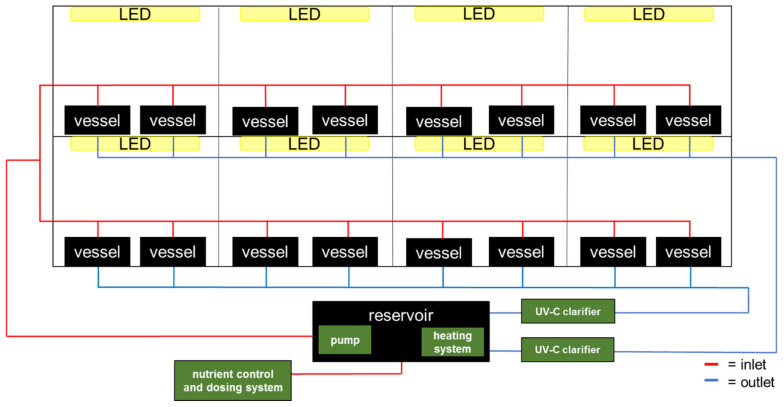
Scheme of the experimental set-up designed as indoor vertical farm (IVF). Black boxes depict the cultivation vessels for the duckweeds and the nutrient solution reservoir, yellow boxes depict the LEDs and green boxes depict the necessary technology to run the re-circulating system. Red lines indicate the nutrient solution inlet and blues lines the outlet.

**Figure 2:**
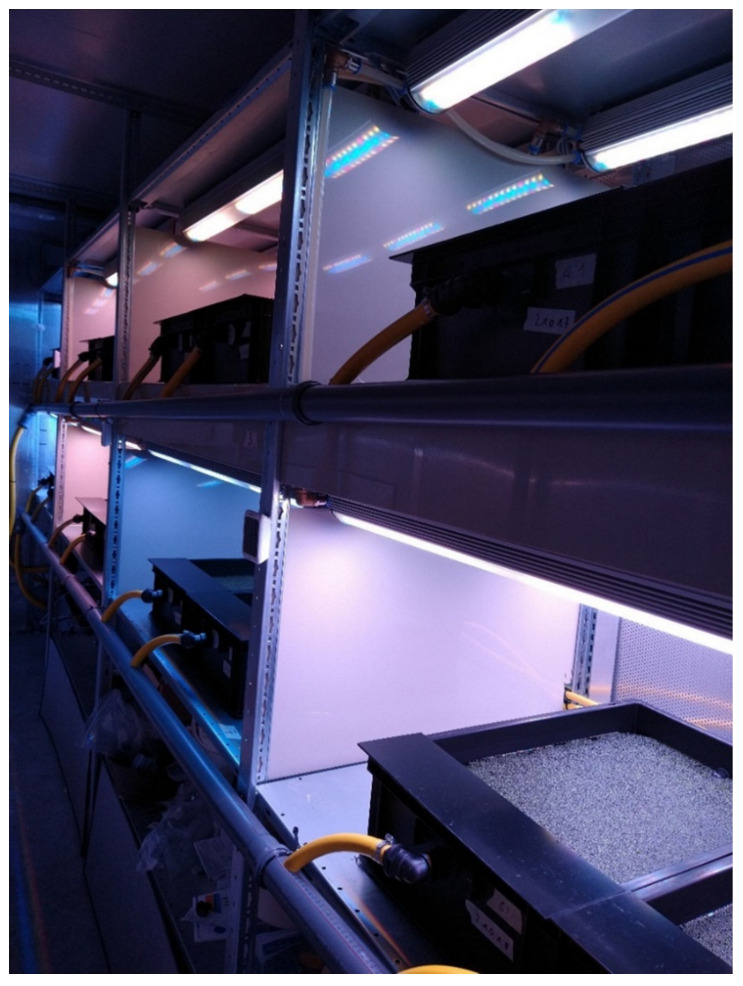
Experimental set-up in the container at the Osnabrück University of Applied Sciences, Germany. The light colors of the different spectral treatments are visible.

Experiments were carried out in a container (length × width × height: 5 × 3 × 3 m) at the campus of the University of Applied Sciences Osnabrück, Germany. Trials were conducted in a re-circulating, aquatic IVF (Figure 1 and Figure 2).

It consisted of a 90 L reservoir for the nutrient solution connected to all duckweed cultivation vessels via flexible tubes. A submergible and adjustable pump (AquaForte DM-10000 Vario, SIBO BV, Veghel, The Netherlands) was installed at the bottom of the reservoir to create a continuous flow between reservoir and cultivation vessels. A nutrient control and dosing system (Pro Controller and PeriPods, Bluelab Corporation Ltd., Tauranga, New Zealand) added the required liquid fertilizers from stock solutions to the tap water in the reservoir. A heating system (Super Fish Smart Heater 500 W, Aquadistri BV, Klundert, The Netherlands) was installed at the bottom of the reservoir to keep a constant water temperature. The vessels (56 cm length × 37 cm width × 10 cm height) used for cultivation were positioned in a two-layer storage rack. On one side (width) of the cultivation vessel, the water inlet, a rectangular pipe leading the water inflow to the bottom of the vessel, was installed. On the opposite side, an outlet was located at 7 cm height. To guarantee no duckweed was lost from the vessel by flowing through the outlet, a wall was installed 7 cm before the outlet. The upper side of the wall was above water level, while the bottom side did not touch the ground of the vessel. This way, the nutrient solution could flow back into the reservoir, while the floating duckweed was hindered from passing the barrier. The net cultivation area per vessel decreased to 0.49 × 0.37 m = 0.1813 m^2^ by applying this method. The unoccupied surface was covered with black PE in order to prevent algae growth in that area. The outlet solution from each of the two storage rack levels was led through UV-C clarifiers (OSAGA UVC36, Fischfarm Otto Schierhölter, Glandorf, Germany) in order to reduce the growth of ubiquitous algae and bacteria.

As light sources, dimmable LEDs with an adjustable spectrum (LED-LE1200-E03W-1-S, DH Licht GmbH, Wülfrath, Germany) were installed 34 cm above the water surface in the vessels. The settings were adjusted with the VisuSpectrum 3.0 software (DH Licht GmbH, Wülfrath, Germany and RAM GmbH Mess- und Regeltechnik, Herrsching, Germany).

### 2.2. Experimental Design

Three different light intensities (50, 100 and 150 µmol m^−2^ s^−1^) were used for the experiments. All of the three spectral treatments contained 20% light at 6500 K (white light), and the remaining 80% were split according to the following red (660 nm)/blue (450 nm) ratio: 70/30, 50/50 and 30/70 (%). This resulted in eight different treatments (Table 1). Light intensities were controlled using a Light Meter LI-250A (LI-COR Biosciences, Lincoln, NE, USA). The photoperiod was set to 12 h of light and 12 h of darkness per day.

Pre-cultivation occurred for three days under above mentioned conditions. Experiments lasted for seven days and were conducted under non-axenic growth conditions. Vessels were placed in the storage rack based on a block design. This storage rack had eight compartments, each containing two LEDs and space for two experimental vessels. Eight treatments, with four replications for each of the two species, were investigated. In total, 16 vessels could be used at a time. Two replicates per light intensity and spectral distribution per species were investigated at the same time. To start with a similar surface coverage of ca. 80% in each vessel, 20 g of *L. minor* and 15 g of *W. hyalina* fresh weight (FW) biomass was placed in each vessel.

The nutrient medium applied mainly consisted of commercially available fertilizers (see Table S1). The nutrient dosing was set to an electrical conductivity (EC) value of 0.6 mS cm^−1^, which corresponds to a nutrient solution of 75-25/10 with the following composition and concentrations (all given in mM): NO_3_^−^-N: 0.76, NH_4_^+^-N: 0.25, PO_4_^3−^: 0.1, K^+^: 0.91, Mg^2+^: 0.13, SO_4_^2−^: 0.32, Ca^+^: 0.22, Cl^−^: 0.34, Fe^3+^: 0.0025, BO_3_^3−^: 0.0005, Mn^2+^: 0.0013, Zn^2+^: 0.001 and Na^+^: 0.08 [28]. When the EC dropped below target value in the time course of cultivation, additional nutrient solution was added until the target EC was reached again.

The pH at the beginning of the experiments was 7.6. The heating system was set to a target value of 24 °C, and the pump was adjusted to a flow rate of 2 L min^−1^.

At the end of the experiments, duckweeds were harvested with a metal sieve, rinsed with tap water, spin-dried for three minutes with a Top Spin Compact (Chal-Tec GmbH, Berlin, Germany) to remove attached water and weighed.

### 2.3. Analytical Methods

#### 2.3.1. Relative Growth Rate

Dry weight (DW) was determined from FW via oven drying at 65 °C for 72 h. At time 0, four samples per species of the same FW as the starting material were used to determine the DW at the beginning of the experiments.

Relative growth rates (RGRs) per day were calculated according to Equation (1) [6], using the values of the DW at the start (t0) and after seven days of cultivation (t7):

RGR = (lnDW_t7_−lnDW_t0_)/(t7−t0) (1)

where RGR is the relative increase in the DW per day (d^−1^).

#### 2.3.2. Crude Protein Content and Relative Protein Yield

Dried samples were ground and homogenized using a laboratory mill and stored for further analysis. The nitrogen content of the dried samples was determined using the Dumas method [29] using an elemental analyzer (FP628, Leco, Saint Joseph, MI, USA), and CPC was calculated using the factor 6.25 [9,30].

The relative weekly yield (RY; g biomass obtained after one week of cultivation starting with 1 g) was calculated from the RGR using Equations (2) and (3):

lnDW_t7_ = lnDW_t0_ + RGR·(t7−t0) (2)

RY = exp(lnDW_t7_) (3)

The RY was further used to calculate the relative protein yield (RPY; g protein week^−1^ m^−2^) by multiplying it with the crude protein content (CPC) and extrapolating it to one square meter, according to Equation (4):

RPY = RY × CPC/(0.1813 m^2^ × 100) (4)

where 0.1813 m^2^ is the cultivation area of the vessels used in the experiments.

#### 2.3.3. Chlorophyll a

The chlorophyll a content was determined according to DIN 38409-60:2019-12 [31], using ethanol (ω(EtOH) = 90%) as a solvent. Four replicates of the starting biomass and four replicates of each treatment at the end of the experiments were analyzed. Laboratory analysis of the chlorophyll a content took place in the dark immediately after the samples were taken according to the following scheme: A net weight of 1.000 ± 0.005 g FW duckweed biomass was placed in 50 mL centrifuge tubes, filled with 10 mL of boiling solvent and homogenized for 60 s using an Ultra-Turrax. The resulting extract was cooled and treated in an ultrasonic bath for 30 min in the dark. Afterwards, the extract was filtered into a 100 mL volumetric flask, filled with ethanol to the calibration mark and homogenized again by shaking. The extract was placed into a glass cuvette. Of the remaining extract, 15 mL was put into a centrifuge tube, added with 100 µL of hydrochloric acid (2 M) and homogenized for the correction of phaeopigments. Both extracts and the pure solvent were finally put into different glass cuvettes and analyzed using a spectrophotometer (Specord 40, Analytik Jena AG, Jena, Germany) at 665 and 750 nm.

The following modified Equation (5) was applied to calculate the chlorophyll a content in the fresh duckweed biomass [31]:

(5) $$\omega_{\mathrm{Chlorophyll}-a}=\left(\left(A_{665v}-A_{750v}\right)-\left(A_{665n}-A_{750n}\right)\right)\cdot \frac{R}{R-1}\cdot \frac{V_{E}}{m_{p}\cdot d\cdot \alpha \cdot 1000}$$

with

ω_Chlorophyll-a_: Chlorophyll a content (mg/g FW);
A_665v_: Absorption of the extract before acidification, measured at 665 nm;
A_750v_: Absorption of the extract before acidification, measured at 750 nm (for the correction of phaeopigments);
A_665n_: Absorption of the extract after acidification, measured at 665 nm;
A_750n_: Absorption of the extract after acidification, measured at 750 nm (for the correction of phaeopigments);
R: Ratio of A_665v_/A_665n_ for pure Chlorophyll-a; R = 1.7;
V_E_: Volume of the extract in milliliters (ml);
m_P_: Net weight of the duckweed biomass sample (g);
d: Thickness of the cuvette (cm); d = 1.

Additionally, the dry matter content of each sample was determined by drying plant material at 105 °C until it reached a constant weight. The chlorophyll a FW content was then multiplied with the dry matter content to calculate the chlorophyll a DW content.

#### 2.3.4. Nutrient Solution

A nutrient solution sample was taken at the start (day 0) and the end (day 7) of the experiments from the reservoir, filtered (MN 619 EH, Machery Nagel GmbH & Co. KG, Düren, Germany) to remove particles and instantly frozen at −18 °C. Nitrate-N and ammonium-N concentrations in these samples were measured according to German standard methods [32,33] with a Lambda 25 UV/VIS Spectrometer (Perkin Elmer, Waltham, MA, USA). Other nutrients were analyzed according to DIN EN ISO 11885:2009-09 with an ICP-OES (ICAP 7400, Thermo Fischer Scientific, Waltham, MA, USA) [34].

### 2.4. Statistics

All data are based on four replicates and are given as mean ± standard deviations. The data were analyzed statistically using one-way ANOVA and Tukey’s post hoc test at 5% significance level, using the software program SPSS 25 (IBM, Armonk, NY, USA).

## 3. Results

### 3.1. Relative Growth Rate

**Figure 3:**
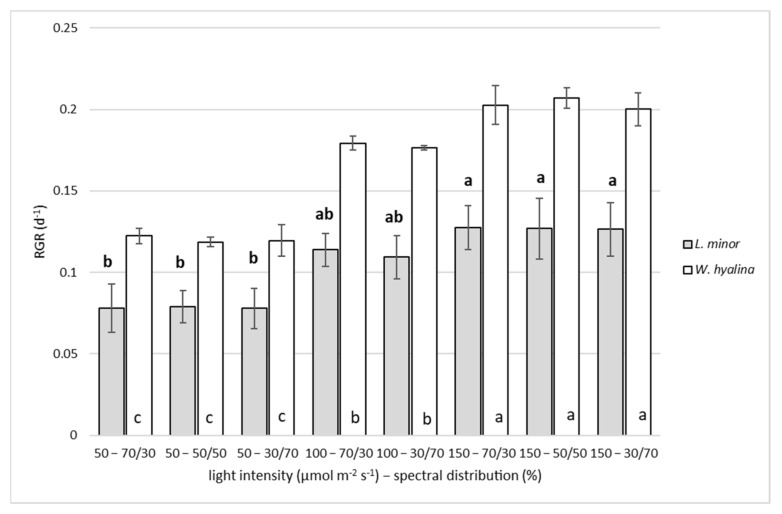
Relative growth rate (RGR; d^−1^), based on dry weight, for Lemna minor (gray shaded columns) and Wolffiella hyalina (white columns). Plants were cultivated for seven days with different light intensities (50, 100 and 150 µmol m^−2^ s^−1^) and spectral distributions (red/blue: 70/30, 50/50 and 30/70%). For the abbreviations used, see Table 1. Number of parallel samples *n* = 4. Different letters indicate significances within a species, based on one-way ANOVA test, Tukey *p* ≤ 0.05. Error bars indicate standard deviations.

Figure 3 shows the RGR based on DW. An increasing light intensity increased the RGR for both species. The highest RGR for *L. minor* was reached at 150–70/30 (0.13 ± 0.013 d^−1^) and for *W. hyalina* at 150–50/50 (0.21 ± 0.01 d^−1^). The minimum values were obtained at 50–30/70 for *L. minor* with an RGR of 0.078 ± 0.012 d^−1^ and at 50–50/50 for *W. hyalina* with an RGR of 0.119 ± 0.003 d^−1^. The percentage increase from the lowest to the highest RGR was 67% for *L. minor* and 76% for *W. hyalina*. The results of all three *L. minor* treatments cultivated at 150 µmol m^−2^ s^−1^ were significantly higher compared to the 50 µmol m^−2^ s^−1^ treatments. *W. hyalina* cultivated at a light intensity of 150 µmol m^−2^ s^−1^ reached significantly higher RGRs than the 100 and 50 µmol m^−2^ s^−1^ treatments. The light spectrum showed no significant impact on the RGR in any treatment.

### 3.2. Crude Protein Content and Relative Protein Yield

The CPC, based on DW, varied in a narrow range between 31.8 ± 0.8% and 32.4 ± 1.2% for *L. minor* and between 39.3 ± 1.0% and 40.0 ± 0.8% for *W. hyalina* for the different treatments. No significant differences in the CPC for the different light intensities and spectral distributions within a species were detected.

**Figure 4:**
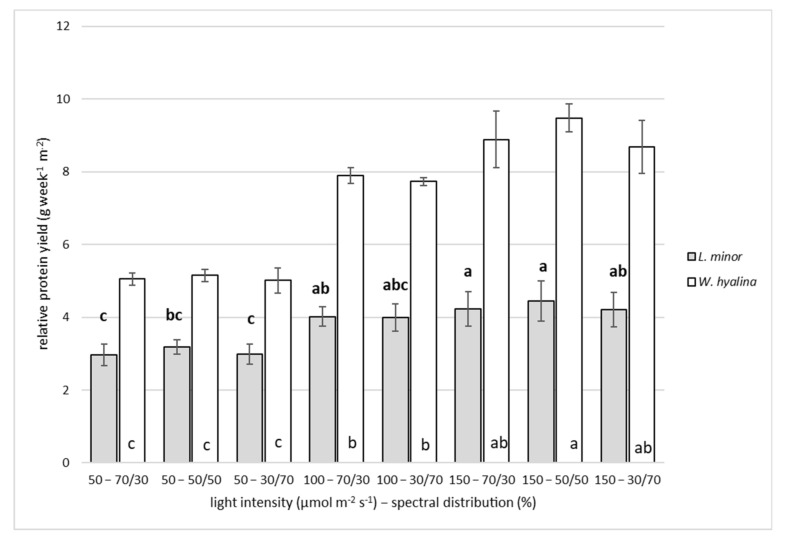
Relative protein yield (RPY; g week^−1^ m^−2^), based on dry weight, for *Lemna minor* (gray shaded columns) and *Wolffiella hyalina* (white columns). For further explanations, see Figure 3.

The RPY in grams per week and m^2^, based on DW, is presented in Figure 4. It ranged from 2.96 ± 0.30 to 4.44 ± 0.55 g week^−1^ m^−2^ (50–70/30 and 150–50/50, respectively) for *L. minor*, while for *W. hyalina*, the range was from 5.01 ± 0.35 g week^−1^ m^−2^ at 50–30/70 to 9.48 ± 0.39 g week^−1^ m^−2^ at 150–50/50. The difference from the lowest to the highest value for *L. minor* was 50%, and for *W. hyalina*, it reached 89%. Higher light intensities resulted in higher relative protein yields. Overall, *W. hyalina* achieved higher RPYs in all treatments compared to *L. minor*. The higher the light intensity, the higher the difference between the species RPYs, meaning that at the highest light intensities (150 µmol m^−2^ s^−1^), *W. hyalina* yielded more protein compared to *L. minor* than at the two lower light intensities. The treatments 50–70/30 and 50–30/70 were significantly lower compared to all other *L. minor* treatments, except for 100–30/70. For *W. hyalina*, all treatments with a light intensity of 50 µmol m^−2^ s^−1^ (50–70/30, 50–50/50 and 50–30/70) were significantly lower compared to the other treatments with higher light intensities. No significant differences, in neither of the two duckweed species, were observed between the different spectral distributions.

### 3.3. Chlorophyll a

**Figure 5:**
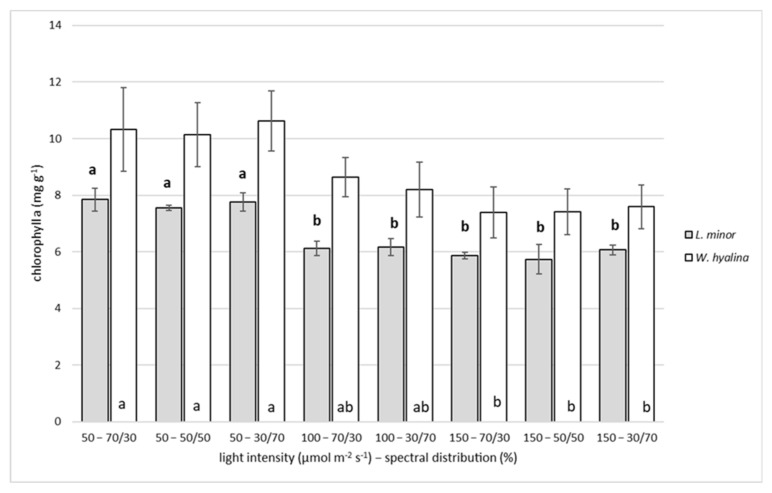
Chlorophyll a content, in mg g^−1^ (based on dry weight), for *Lemna minor* (gray shaded columns) and *Wolffiella hyalina* (white columns). For further explanations, see Figure 3.

The content of chlorophyll a for both species after seven days of experiments ranged between 5.32 ± 0.51 mg g^−1^ and 7.29 ± 0.39 mg g^−1^ for *L. minor* at 150–50/50 and 50–70/30, respectively (Figure 5). The maximum content for *W. hyalina* was 9.98 ± 1.01 mg g^−1^ chlorophyll a, achieved at 50–30/70, while the minimum content (6.83 ± 0.39 mg g^−1^) was obtained at 150–30/70. This corresponded to a decrease of 27% for *L. minor* and 32% for *W. hyalina*.

A significant decline between the treatments of the lowest light intensity (50 µmol m^−2^ s^−1^) and the two higher treatments (100 and 150 µmol m^−2^ s^−1^) can be observed for *L. minor*. For *W. hyalina*, the 150 µmol m^−2^ s^−1^ treatments were significantly lower compared to the 50 µmol m^−2^ s^−1^ treatments. Different light spectra had no significant impact on the chlorophyll a content of both species.

### 3.4. Nutrients

In Table 2, the percentage reduction in different nutrient components in the nutrient medium after seven days of experiments compared to the initial concentration is presented. A percentage increase (shown as negative values) in certain substances was possible due to the EC-based nutrient dosing of the stock solutions.

A strong reduction of more than 80% can be seen for ammonium-N, iron, manganese, zinc, and in case of *L. minor*, also for boron. Nitrate-N was only slightly decreased for *L. minor* (12.8%) and showed a minor increase for *W. hyalina*. Similar results were also observed for potassium. An increase in magnesium, sulfur and calcium occurred for both species.

Compared to the start of experiments, the pH showed a minor increase with an average value of 7.8 for the *L. minor* experiments and 7.9 for the *W. hyalina* experiments.

## 4. Discussion

### 4.1. Relative Growth Rate

The RGR determined in our study differed between both investigated species and growth conditions. An increase in light intensity from 50 to 150 µmol m^−2^ s^−1^ significantly increased the RGR of *L. minor* and *W. hyalina*. Our data agree with other published investigations. Paolacci et al. [26] reported that increasing light intensities between 6 and 1000 µmol m^−2^ s^−1^ increased the RGR of *L. minor* and *L. minuta* cultivated in sterile growth rooms at 20 °C with a light:dark cycle of 16:8 h. At light intensities below 40 µmol m^−2^ s^−1^, no significant differences were detected between the RGR of both species, while above 90 µmol m^−2^ s^−1^, *L. minuta* had significantly higher RGRs than *L. minor*. The latter reached an RGR of 0.26 d^−1^ when grown at 150 µmol m^−2^ s^−1^. This was higher compared to our result, but cultivation conditions varied, which might provide a possible explanation for this difference.

At comparatively low light intensities between 30 to 105 µmol m^−2^ s^−1^, *L. aequinoctialis* reached an RGR of 0.19 d^−1^ at the highest light intensity, when cultivated in monoculture, while *L. punctata* and *Spirodela polyrhiza* reached 0.18 d^−1^ and 0.15 d^−1^ under the same growth conditions, respectively [35]. Increasing light intensity and photoperiod increased growth rate, biomass and starch production in *L. aequinoctialis*. Considering the costs for lighting, an optimum regarding those factors was reached at 110 µmol m^−2^ s^−1^ [24]. A sevenfold increase in light intensity (from 100 to 700 µmol m^−2^ s^−1^) resulted in a 25% greater RGR of *L. gibba* [23]. This increase in RGR was lower compared to *L. minor’s* RGR increase of 67% and *W. hyalina*’s increase of 76% at a 200% greater light input in our study.

The maximum obtained RGRs of 0.13 d^−1^ for *L. minor* and 0.21 d^−1^ for *W. hyalina* in the presented study are lower compared to the highest achieved values of 0.42 d^−1^ and 0.52 d^−1^ for the same clones, respectively, grown under sterile conditions in batch cultures [6]. However, under non-axenic conditions, certain cultivation adaptations due to inhibiting factors, such as algae or fungus growth, need to be considered [36,37]. A highly diluted growth medium, comparatively low light intensities and a moderate temperature were applied in our re-circulating IVF for non-axenic duckweed cultivation. Regarding the investigation of Petersen et al. [28], the same nutrient medium with a dilution of 10% resulted in an RGR of 0.21 d^−1^ for *W. hyalina*. This is in exact agreement with the results of the current study.

In contrast, other studies reported that different light intensities had no significant impact on the RGR of duckweed species. The RGR of *Lemna minor* grown on synthetic dairy wastewater did not increase with increasing light intensities between 50 and 850 µmol m^−2^ s^−1^ [38]. *Lemna gibba* reached constant high growth rates under different light intensities between 50 and 1000 µmol m^−2^ s^−1^; however, higher intensities led to increasing zeaxanthin levels. This way, a large fraction of the absorbed light was dissipated non-photochemically [39].

The light spectra in the presented experiments had no significant impact on any investigated parameters for both species. However, it has to be kept in mind that in this study, pure red or blue light was never used. There was always a white light background of the light intensity of 20%, and the ratios between blue and red light were never higher than 70:30%.

Up to now, only a few investigations concerning this parameter have been carried out regarding duckweed RGR. *Landoltia punctata* cultivated under fluorescent white light, blue LED and white LED at 110 µmol m^−2^ s^−1^ showed no significant RGR differences [40]. There was also no significant difference in the RGR of *S. polyrhiza* when cultivated at 60 µmol m^−2^ s^−1^ using red and blue LEDs (660 and 460 nm, respectively) [41], which is in agreement with our results. Xu et al. [42] described that the application of red and blue light at the same time can be absorbed by plants more efficiently compared to other spectra and resulted in high photosynthetic efficiency. *Spirodela polyrhiza* cultured in eutrophic medium reached a significantly higher total biomass yield when a red:blue ratio of 2:1 or 4:1 at a light intensity of 110 µmol m^−2^ s^−1^ was applied compared to monochromatic (450, 630 or 660 nm) or fluorescent light sources at the same intensities.

### 4.2. Crude Protein Content and Relative Protein Yield

The presented crude protein contents for both species showed no significant difference between the tested light scenarios. This is in contrast to the results reported by Stewart et al. [39], who showed that the protein content of *L. gibba*, cultivated at 50 and 1000 µmol m^−2^ s^−1^, increased from 25% to 46%, respectively. A protein content increase from 1.5% to 2% (based on FW) was observed for *L. minor* when cultivated on synthetic dairy wastewater at a light intensity of 850 µmol m^−2^ s^−1^ compared to 50 µmol m^−2^ s^−1^. In C3 plants, such as duckweed, higher light intensities induce the increased production of Rubisco, a soluble protein [38]. A small increase in light intensity (from 200 to 400 µmol m^−2^ s^−1^) only slightly increased the percentage of activated Rubisco in *S. polyrhiza* [43]. This could be an explanation for the relatively stable crude protein contents in our study, as the light intensity only slightly increased from 50 to 150 µmol m^−2^ s^−1^. A more substantial increase in light intensity, as described above, will lead to rising protein contents.

The crude protein contents in the presented experiments were rather high considering the low nutrient concentration and the low light intensities, especially regarding *W. hyalina*. Appenroth et al. [9] reported a crude protein content of 35% for *W. hyalina* and 25% for *L. minor*. These duckweeds were cultivated with a modified Schenk–Hildebrandt medium at 100 µmol m^−2^ s^−1^ continuous white light. In another experiment, the highest values for crude protein of the three species *L. aequinoctialis*, *L. punctata* and *S. polyrhiza* (33.7, 32.3 and 36.8%, respectively), were reached at 105 µmol m^−2^ s^−1^ using a one-tenth strength Hoagland solution [35]. Petersen et al. [28] reached CPCs of 32.4% for *L. minor* and 35.3% for *W. hyalina* using a stationary system with the same nutrient solution as applied in these experiments. Wheeler at al. [44] assumed that a continuous supply of nitrogen caused higher protein levels in different crops (wheat, lettuce, potato and soybean) grown in a re-circulating hydroponic system compared to the same field-grown crops. Such a mechanism might also be responsible for the CPC increase in *W. hyalina*, cultivated in the re-circulating system compared to the stationary system.

A red:blue ratio of 1:2 can increase starch yield significantly, while a higher portion of the red spectrum under eutrophic conditions caused a strong inductive effect on turion formation in *S. polyrhiza* [42]. This is contrary to data reported by Zhong et al. [41], who detected an increased starch accumulation for the same species using red light, while blue light promoted protein accumulation. In *W. arrhiza*, using irradiation with wavelengths corresponding to white, red and blue light, no significant differences in amino acid concentrations of the soluble protein were detected [45]. These results fit to our findings that the spectral distribution as applied did not significantly influence CPC.

The protein productivity, given as RPY, was lower for *L. minor* compared to *W. hyalina*. The species *L. minor* reached a maximum of 4.44 ± 0.55 g week^−1^ m^−2^ at 150–50/50 and *W. hyalina* of 9.48 ± 0.39 g week^−1^ m^−2^ for the same treatment. This extrapolates to 2.31 and 4.93 t of pure protein per year and hectare, respectively. In the literature, a wide range of productivities are reported. For *L. minor* and *W. hyalina*, 28.8 and 34.7 g week^−1^ m^−2^, respectively, were reached using the same nutrient solution in a stationary system with smaller vessels [28]. Mohedano et al. [46] reported a protein productivity of 24 t year^−1^ ha^−1^ (ca. 46 g week^−1^ m^−2^) for duckweeds. Chakrabarti et al. [47] reached a biomass yield of 703 kg month^−1^ ha^−1^ (ca. 17.5 g week^−1^ m^−2^) for *L. minor*. Regarding protein content of 27.1% for duckweed grown on an inorganic fertilizer-based solution, the protein productivity resulted in 4.74 g week^−1^ m^−2^. Comparing these values to soybean with a yield of ca. 3 t year^−1^ ha^−1^ and a protein content of 40% [48], the protein productivity of 1.2 t year^−1^ ha^−1^ was considerably lower compared to any duckweed protein productivity projection.

### 4.3. Chlorophyll

The chlorophyll a content for both species was investigated as a parameter to indicate a possible color changes in the plants at different light conditions. It decreased with increasing light intensity. This negative correlation was also found for other duckweed species [23,26,38,39,49]. *L. minor* had higher total chlorophyll contents for all investigated light intensities (6 to 1000 µmol m^−2^ s^−1^) than *L. minuta*, reaching up to ca. 1.4 mg g^−1^ of fresh biomass at the lowest light intensity [26]. *Lemna gibba* contained ca. 250 µmol m^−2^ of chlorophyll a and b at 50 µmol m^−2^ s^−1^ and ca. 300 µmol m^−2^ at 100 µmol m^−2^ s^−1^ [23,39]. The reduction in chlorophyll at high light intensities is an acclimation strategy, protecting the plant against light-induced damage due to photo oxidation [50]. Contrarily, high chlorophyll contents at low light intensities ensure maximal light absorption. Such plants are usually associated with shade tolerance [26].

The different investigated spectral distributions had no significant impact on both species’ chlorophyll content. This has also been shown by Zhong et al. [41], who obtained no significant differences in *S. polyrhiza*, when cultivated under red, blue and white light. This missing effects of the light quality in our experiment might be also caused by the use of mixed light quality.

## 5. Conclusions

The duckweed cultivation system applied in our experiments was a small-scale, experimental prototype of a re-circulating, aquatic IVF and specifically designed and built for conducting scientific experiments. In the literature, only a theoretical approach [13], but no practical application of an IVF for duckweed cultivation has been described, neither on a small scale for experiments nor on a large scale for biomass production. This small-scale, re-circulating IVF for scientific experiments fits the criteria for a plant factory with artificial lighting regarding structure, functionality and operation goals in most aspects [16]. The results of the present study underline the idea that the cultivation of duckweeds in such a system under non-sterile conditions is feasible and might be up-scaled for mass production.

The applied system for nutrient control and dosing is based on EC values. When the actual EC values fell below the target EC, the dosing system pumped stock solution into the reservoir until the target value was reached again. This is a well-established system for nutrient dosing used in many different hydroponic applications [16,51]. However, when used in re-circulating systems, the disadvantages become obvious. An imbalance between nutrient composition of the stock solutions and actual nutrient uptake by the plants can cause increasing concentrations of certain substances in re-circulating systems, as happened in our experiments. The longer a re-circulating system runs, the greater the imbalances will become. A depletion of nutrients, such as ammonium, nitrate, sodium or magnesium, can cause reduced RGR, CPC or RPY in duckweed due to non-optimal nutrient ratios [28,52]. In the case of nitrogen, duckweeds preferentially take up ammonium over nitrate [53]. An adaptation of the stock solutions to the actual plants’ demands is difficult due to plant physiological and technical reasons. Many crops have changing demands at different plant development stages. Additionally, the dosing pumps must work precisely, when dosing more than one stock solution, to keep the nutrient ratio at a given target level. The use of stationary, on-line, ion-selective sensors [54], ion-sensitive field-effect transistors [55] or mid-infrared sensors [56] might be options to solve the problem in the future, but to date, not all relevant nutrients for plant growth can be measured. Relevant aspects regarding the application in hydroponics are the frequency and complexity of sensor calibrations, lifespan and costs as well as the stability, selectivity and drift of these technologies [54,55,57]. The readiness levels of these technologies currently vary, but new components and membranes will improve the coming product generations [55].

**Figure 6:**
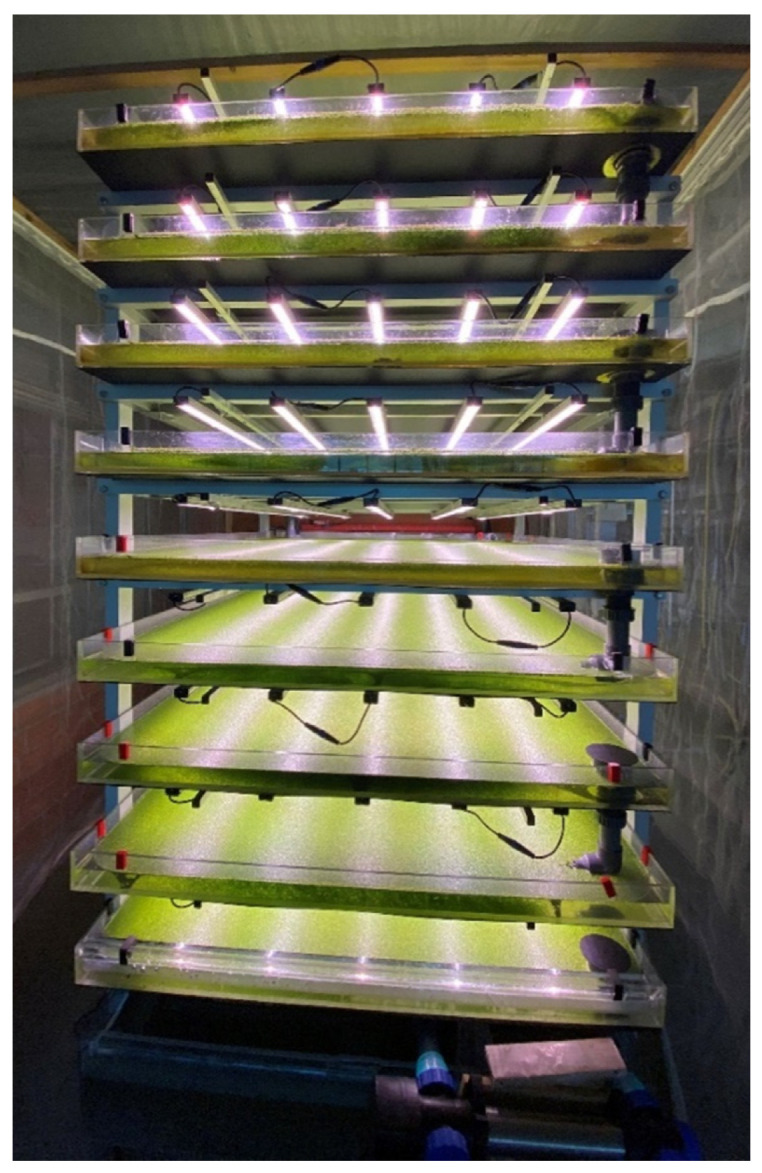
Large scale, re-circulating indoor vertical farm (IVF) or duckweed biomass cultivation at the University of Applied Sciences Osnabrück, Germany.

To gain more data about the behavioral pattern of duckweed in re-circulating systems, longer-lasting experiments investigating a broad range of abiotic, and in the case of non-sterile experiments, also biotic, parameters are needed. Nonetheless, the findings and experiences of our study were already successfully implemented into the operation of a large scale, re-circulating, aquatic IVF for duckweed biomass cultivation (Figure 6).

## Figures and Tables

**Table 1:** Applied light intensities and red/blue ratios (spectral distributions) in the experiments as well as the corresponding treatment abbreviation, as used throughout the text.

Light Intensity	Red/Blue Ratios	Treatment Abbreviation
50	70/30	50–70/30
50	50/50	50–50/50
50	30/70	50–30/70
100	70/30	100–70/30
100	30/70	100–30/70
150	70/30	150–70/30
150	50/50	150–50/50
150	30/70	150–30/70

**Table 2:** Percentage reduction in nutrient solution substances for *L. minor* and *W. hyalina*, based on one solution sample taken at the beginning and the end of experiments from the reservoir. Duckweeds were separately cultivated for seven days in a re-circulating, aquatic system. Negative values indicate an increase in the corresponding substance due to EC-based nutrient dosing.

Substance	*L. minor*	*W. hyalina*
NH_4_^+^-N	97.2	97.7
NO_3_^−^-N	12.8	−6.6
PO_4_^3−^	52.8	26.6
K^+^	7.9	−1.4
Mg^2+^	−7.9	−22.6
SO_4_^2−^	−8.0	−11.7
Ca^+^	−8.1	−10.1
Fe^3+^	95.6	94.5
BO_3_^3−^	84.8	2.2
Mn^2+^	80.3	98.6
Zn^2+^	84.2	89.7
Na^+^	29.1	23.7

## Data Availability

All data are available within the manuscript or Supplementary Material.

## Supplementary Materials

The following supporting information can be downloaded at: https://www.mdpi.com/article/10.3390/plants11081010/s1, Table S1. Formulation of seven stock solutions (g L^−1^) for five different nitrate-N to ammonium-N ratios.
