# Supplementary material for: Influence of Light Intensity and Spectrum on Duckweed Growth and Proteins in a Small-Scale, Re-Circulating Indoor Vertical Farm
Source: Plants (Basel). 2022 Apr 7;11(8):1010. doi: 10.3390/plants11081010 (PMC9030439; doi:10.3390/plants11081010)
Supplement: Supplementary file 1 [file plants-11-01010-s001.zip › Table S1.pdf]

Table S1. Formulation of seven stock solutions (g L^{-1}) for five different nitrate-N to ammonium-N ratios ([100-0], [75-25], [50-50], [25-75], [0-100]), based on the N-medium.

Stock solution	Product name	Main components	[100-0] (g L^{-1})	[75-25] (g L^{-1})	[50-50] (g L^{-1})	[25-75] (g L^{-1})	[0-100] (g L^{-1})
1	Calcinit	NO_3^--N, NH_4^+-N, Ca^+	47.2	35.4	23.6	11.8	0
1	Krista K Plus	NO_3^--N, K^+	161.8	121.3	80.9	40.4	0
2	NH_4Cl	NH_4^+-N, Cl^-	0	0	26.7	53.5	80.2
3	OCI Granular 2	NH_4^+-N, SO_4^{2-}	0	33	33	33	33
4	KCl	K^+, Cl^-	0	29.8	59.6	89.5	119.3
4	$CaCl_2$ * 2 H_20	Ca^+, Cl^-	0	7.4	14.7	22.1	29.4
5	Krista MKP	PO_4^{3-}, K^+	27.2	27.2	27.2	27.2	27.2
6	Epso Combitop	Mg^{2+}, SO_4^{2-}, Mn^{2+}, Zn^{2+}	49.3	49.3	49.3	49.3	49.3
6	Borax	BO_3^{3-}	0.06	0.06	0.06	0.06	0.06
6	Mangaan	Mn^{2+}, SO_4^{2-}	0.44	0.44	0.44	0.44	0.44
6	$MoNa_2O_4$ * 2 H_20	MoO_4^{2-}, Na^+	0.02	0.02	0.02	0.02	0.02
7	Ferty 72	Fe^{3+}	2.2	2.2	2.2	2.2	2.2
